# Identifying potential drug targets for varicose veins through integration of GWAS and eQTL summary data

**DOI:** 10.3389/fgene.2024.1385293

**Published:** 2024-05-15

**Authors:** Yu Cui, Mengting Hu, He Zhou, Jiarui Guo, Qijia Wang, Zaihua Xu, Liyun Chen, Wancong Zhang, Shijie Tang

**Affiliations:** ^1^ Shantou University Medical College, Shantou, Guangdong, China; ^2^ Research Center of Translational Medicine, Second Affiliated Hospital of Shantou University Medical College, Shantou, Guangdong, China; ^3^ Plastic Surgery Institute of Shantou University Medical College, Shantou, Guangdong, China; ^4^ Shantou Plastic Surgery Clinical Research Center, Shantou, Guangdong, China; ^5^ Department of Plastic Surgery and Burns Center, Second Affiliated Hospital, Shantou University Medical College, Shantou, Guangdong, China

**Keywords:** varicose veins, drug targets, Mendelian randomization analysis, colocalization analysis, genome-wide association studies, expression quantitative trait locus

## Abstract

**Background:**

Varicose veins (VV) are a common chronic venous disease that is influenced by multiple factors. It affects the quality of life of patients and imposes a huge economic burden on the healthcare system. This study aimed to use integrated analysis methods, including Mendelian randomization analysis, to identify potential pathogenic genes and drug targets for VV treatment.

**Methods:**

This study conducted Summary-data-based Mendelian Randomization (SMR) analysis and colocalization analysis on data collected from genome-wide association studies and cis-expression quantitative trait loci databases. Only genes with PP.H4 > 0.7 in colocalization were chosen from the significant SMR results. After the above analysis, we screened 12 genes and performed Mendelian Randomization (MR) analysis on them. After sensitivity analysis, we identified four genes with potential causal relationships with VV. Finally, we used transcriptome-wide association studies and The Drug-Gene Interaction Database data to identify and screen the remaining genes and identified four drug targets for the treatment of VV.

**Results:**

We identified four genes significantly associated with VV, namely, *KRTAP5-AS1* [Odds ratio (OR) = 1.08, 95% Confidence interval (CI): 1.05–1.11, *p* = 1.42e-10] and *PLEKHA5* (OR = 1.13, 95% CI: 1.06–1.20, *p* = 6.90e-5), *CBWD1* (OR = 1.05, 95% CI: 1.01–1.11, *p* = 1.42e-2) and *CRIM1* (OR = 0.87, 95% CI: 0.81–0.95, *p* = 3.67e-3). Increased expression of three genes, namely, *KRTAP5-AS1*, *PLEKHA5*, and *CBWD1*, was associated with increased risk of the disease, and increased expression of *CRIM1* was associated with decreased risk of the disease. These four genes could be targeted for VV therapy.

**Conclusion:**

We identified four potential causal proteins for varicose veins with MR. A comprehensive analysis indicated that *KRTAP5-AS1*, *PLEKHA5*, *CBWD1*, and *CRIM1* might be potential drug targets for varicose veins.

## 1 Introduction

Varicose veins (VV) is a common chronic venous disease affecting approximately one-third of the global population (Oliveira et al., 2021). VV can manifest with a wide range of clinical presentations, ranging from asymptomatic to severe symptoms, including edema, pigmentation changes, eczema, lipodermatosclerosis, atrophie blanche, and healing or active venous ulcers (Youn and Lee, 2019). The economic burden of VV on healthcare systems is substantial (Malgor and Labropoulos, 2012). Current treatment approaches primarily involve pharmacotherapy, which is not always universally effective, necessitating more targeted therapeutic methods (Belramman et al., 2019).

The incidence of VV is influenced by various factors, such as genetic susceptibility, environmental factors, hormones, endothelial dysfunction, activation of inflammatory cells and molecules, and disruption of cytokines and matrix metalloproteinase balance (Raffetto, 2018). Observational epidemiological studies are prone to confounding, reverse causation, and various biases, which limit a deep understanding of the disease’s pathogenesis and treatment targets. However, Mendelian randomization (MR) methods, which are based on the random distribution of genetic polymorphisms, simulate the effects of randomized controlled trials and can eliminate the influence of confounding factors. In the context of VV research, this implies a more accurate assessment of the causal relationship between the drug targets and disease while excluding factors that could interfere with the results (Smith and Hemani, 2014). There are also many studies that use machine learning and other methods to explore drug targets (Yang et al., 2021; Yang et al., 2022).

MR employs genetic variants as instrumental variables to estimate the causal effect of an exposure on the outcomes. It has been widely applied in other disease studies and helped successfully identify therapeutic targets for various diseases (Georgakis and Gill, 2021; Cao et al., 2023). However, there is limited research on employing MR methods to explore potential drug targets for VV. Therefore, to gain a deeper understanding of the pathogenesis of VV and identify more effective treatment approaches, further MR studies are needed to evaluate the drug targets for VV. This will help eliminate factors that could interfere with the results and offer new perspectives and methods for the treatment of VV.

Conceptually, single nucleotide polymorphisms (SNPs) are randomly distributed and not influenced by environmental factors, making them an ideal tool for causal inference. MR is a form of instrumental variable analysis that primarily utilizes SNPs as genetic instruments to estimate the causal effect of an exposure (in this case, circulatory proteins) on the outcomes (Byrska-Bishop et al., 2022). MR has been successfully used in previous studies to identify biomarkers and treatment targets for various diseases, including aortic aneurysms (Chen et al., 2022), multiple sclerosis (Lin et al., 2023b), and breast cancer (Wang et al., 2023).

In the MR analysis of drug targets, cis-expression quantitative trait loci (cis-eQTLs) located in the drug target gene regions are often considered proxies that act as regulators of gene expression. Previous research has identified potential pathogenic proteins for VV, such as IRF3, LUM, POSTN, RSPO3, and SARS2, through a drug target MR analysis that focused on 2,004 plasma proteins (Lin et al., 2023a). However, this study had significant limitations, and the analysis of drug targets for VV remains insufficient, with numerous potential drug targets awaiting discovery. To address this research gap, our study focuses on conducting drug-target research with genes as the exposure factor for VV. Unlike previous studies that used proteins as the exposure factor, we explored the role of genes in the development of VV and as potential drug targets. Research using genes as the exposure factor has several advantages. First, genes have stable genetic characteristics, and they are unaffected by environmental factors, allowing for a more accurate assessment of the causal relationship between genes and VV (Shah et al., 2020). Second, using genes as the exposure factor can provide more direct information and help reveal potential pathogenic mechanisms and drug targets for VV (Crous-Bou et al., 2016). By exploring VV’s potential drug targets through genes as the exposure factor, we aim to offer new perspectives and methods for the treatment and prevention of VV.

In this study, our goal was to identify potential pathogenic genes for VV. We conducted summary-data-based Mendelian randomization (SMR, 2022), MR, and transcriptome-wide association study (TWAS, 2022) analyses by combining eQTLs identified in blood with independent genome-wide association study (GWAS) datasets for varicose veins.

## 2 Materials and methods

### 2.1 Datasets

#### 2.1.1 GWAS

We obtained summary-level GWAS data on VV from the FinnGen consortium, which comprise 17,027 cases with VV and 190,028 controls. To the best of our knowledge, these data constitute the most recent GWAS findings, encompassing the largest number of VV cases to date. The overarching objective of FinnGen is to accumulate and meticulously analyze the genome and national health register data from 500,000 Finnish individuals (Kurki et al., 2023).

For external replication with an independent sample cohort, we used the summary statistics from the UK Biobank. The UK Biobank is a large-scale biomedical database that encompasses genetic and health information of more than 500,000 participants in the United Kingdom. This resource provides a valuable platform for researchers and offers extensive data on various health conditions, lifestyle factors, and genetic profiles. In our study, the UK Biobank dataset included 10,044 patients with VV and 452,966 controls and contributed to the robustness and generalizability of our findings (Fry et al., 2017; Hemani et al., 2018).

#### 2.1.2 eQTL

In our study’s context of drug development, we focused on cis-eQTLs that were in proximity to the target gene. These cis-eQTLs for the SMR analysis were sourced from the eQTLGen Consortium and the eQTL meta-analysis conducted on peripheral blood samples from a cohort of 31,684 individuals (Võsa et al., 2021). These selected cis-eQTLs served as valuable inputs for our SMR, colocalization (Giambartolomei et al., 2014), and MR analyses.

For the TWAS analysis of the eQTL data, we used the Genotype-Tissue Expression (GTEx) project v8 European whole blood dataset. GTEx is a notable biomedical research endeavor dedicated to comprehending the diversity in human gene expression across diverse tissues and organs to elucidate its intricate connections with genotype. Given our specific emphasis on VV, we meticulously extracted comprehensive eQTL results exclusively from the whole blood samples within the GTEx dataset (GTEx Consortium, 2020).

#### 2.1.3 DGIdb

The Drug Gene Interaction Database (DGIdb) is as a comprehensive drug-gene interaction network resource. It amalgamates various data sources that elucidate the interactions between drugs and genes (Wagner et al., 2016) as well as the pharmaceutical relevance of genes. In this study, we used the DGIdb for the identification of candidate gene targets for gene therapy from the previously identified genes.

### 2.2 SMR analysis

**FIGURE 1:**
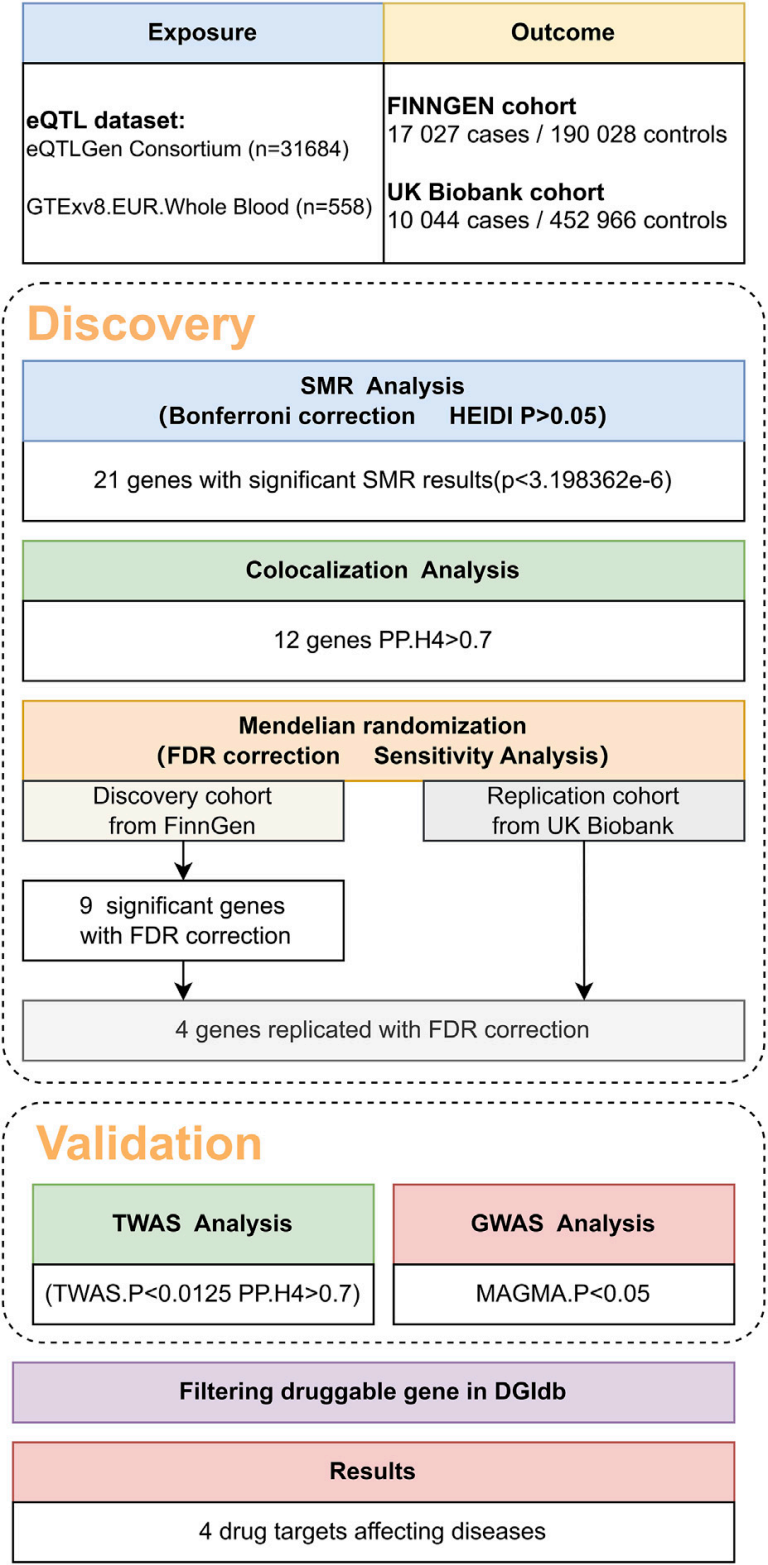
Overview of the study design.

A flow diagram summarizing the methodology is shown in Figure 1. We conducted summary data-based Mendelian randomization (SMR) and heterogeneity in dependent instruments (HEIDI) tests analyses on cis regions using the SMR software (version 1.03) (SMR, 2022). The methodologies for SMR analysis are detailed in the original work by (Zhu et al., 2016). In brief, SMR analysis uses a well-established MR approach. This technique employs a SNP at a prominent cis-eQTL as an instrumental variable (IV). The summary-level eQTL data act as the exposure, and the GWAS data for a specific trait serve as the outcome. The primary objective is to explore a potential causal or pleiotropic association, wherein the same causal variant is shared between gene expression and the trait.

It is crucial to acknowledge that the SMR method lacks the ability to distinguish between a causal association, in which gene expression causally influences the trait, and a pleiotropic association, in which the same SNP affects both gene expression and the trait. This limitation arises due to the single IV in the MR method, which cannot differentiate causality from pleiotropy. Nevertheless, the HEIDI test can make this distinction by discerning causality and pleiotropy from linkage. Linkage denotes cases in which two different SNPs in linkage disequilibrium (LD) independently impact gene expression and the trait. Although less biologically intriguing than causality and pleiotropy, the HEIDI test brings clarity in such scenarios.

For the SMR tests, statistical significance was set at a *p*-value less than 3.198362 × 10^-6 (Bonferroni correction of 0.05 divided by 15,633, the number of results in the eQTL data). For the HEIDI test, a *p*-value below 0.05 was considered significant and suggesting that the observed association was attributable to linkage (Zhu et al., 2016).

### 2.3 Colocalization analysis

We conducted colocalization analysis using the coloc package within the R software environment (version 4.0.3) (Giambartolomei et al., 2014). The purpose of colocalization analysis is to evaluate whether SNPs associated with both gene expression and phenotype at a given locus are indeed shared causal variants, indicating the “colocalization” of gene expression and the phenotype. The analysis, implemented through colocalization analysis, computes posterior probabilities (PPs) for five hypotheses: 1) H0, indicating no association with either gene expression or the phenotype; 2) H1, denoting an association solely with gene expression; 3) H2, signifying an association exclusively with the phenotype; 4) H3, suggesting an association with both gene expression and the phenotype through independent SNPs; and 5) H4, implying an association with both the gene expression and phenotype through shared causal SNPs. A substantial PP for H4 (PP.H4 above 0.70) strongly indicates the existence of shared causal variants influencing both gene expression and the phenotype (Giambartolomei et al., 2014).

### 2.4 MR analysis

The two-sample MR analysis was chosen due to its robustness in assessing causality in observational data, by using SNPs as IVs, this method avoids the behavioral and environmental factors that influence epidemiological studies, reducing potential bias in observational studies. To perform the two-sample MR analysis (Hemani et al., 2018; Two Sample, 2022), we used the TwoSampleMR R package (version 0.5.6). The application of the two-sample MR framework requires the use of two distinct datasets. In this study, genetic instruments, specifically cis-eQTL, were employed as the exposures, and GWAS data served as the outcome trait data. The MR methodology evaluates the association between gene expression and diseases or traits by leveraging genetic variants linked to gene expression as the instrumental variables (exposure) and GWAS as the outcomes. MR allows for the exploration of whether changes in gene expression causally influence diseases or traits. For instruments represented by a SNP, the Wald ratio was applied. In the cases in which the instruments comprised multiple SNPs, the inverse variance-weighted MR approach was implemented.

The MR analysis was exclusively conducted for the results of the SMR and colocalization analyses. We applied the false discovery rate (FDR) method to correct the *p* values. The analysis of pleiotropy employed the MR_pleiotropy_test function, where a *p*-value less than 0.05 was considered to indicate the presence of pleiotropy, and a *p*-value greater than 0.05 indicated its absence. Significance parameters were set to *p* < 5 × 10^-8 for genome-wide significance; the linkage disequilibrium parameter (r^2) was set to 0.1, and the genetic distance was set to 10 Mb.

### 2.5 TWAS analysis

TWAS is a research approach employed to establish connections between the gene expression levels and intricate networks governing gene expression. By scrutinizing the extensive transcriptomic datasets, researchers can discern distinct gene expression patterns across varied biological conditions. This method enables the identification of pivotal genes linked to specific physiological processes or diseases while elucidating intricate gene interactions. Through these investigations, essential genes crucial to distinct biological processes are pinpointed, revealing their regulatory networks and potential signaling pathways (Gusev et al., 2016).

In this study, a TWAS analysis was conducted on autosomal chromosomes using Fusion software (TWAS), following the default parameters specified in the fusion guidelines (Gusev et al., 2016). To assess colocalization, we employed the coloc R package for genes that achieved transcriptome-wide significance and were located within a 1.5 Mb range. This Bayesian methodology calculates posterior probabilities (PP), representing the likelihood of shared causal variants within a locus for two outcomes. The analytical approach employed in this study distinguishes associations influenced by horizontal pleiotropy (a solitary causal SNP impacting both transcription and VV; posterior probability PP4) from those arising due to linkage (two causal SNPs in LD affecting transcription and VV independently; posterior probability PP3) (Giambartolomei et al., 2014).

### 2.6 GWAS analysis

The Multimarker Analysis of Genomic Annotation (MAGMA) serves as a versatile instrument for gene examination and comprehensive gene-set analysis of GWAS data (MAGMA, 2022). It facilitates the analysis of raw genotype data and summary SNP *p*-values derived from prior GWAS endeavors or meta-analyses. MAGMA utilizes a multiple regression model for assessing the combined effect of several SNPs assigned to a specific gene (±10 kb) (Leeuw et al., 2015). The Phase 3 dataset from the European population of the 1000 Genomes Project is employed as the reference panel for computing linkage disequilibrium (LD) (The 1000 Genomes Project Consortium et al., 2015).

## 3 Result

### 3.1 SMR analysis and colocalization for preliminary identifying potential genes

**FIGURE 2:**
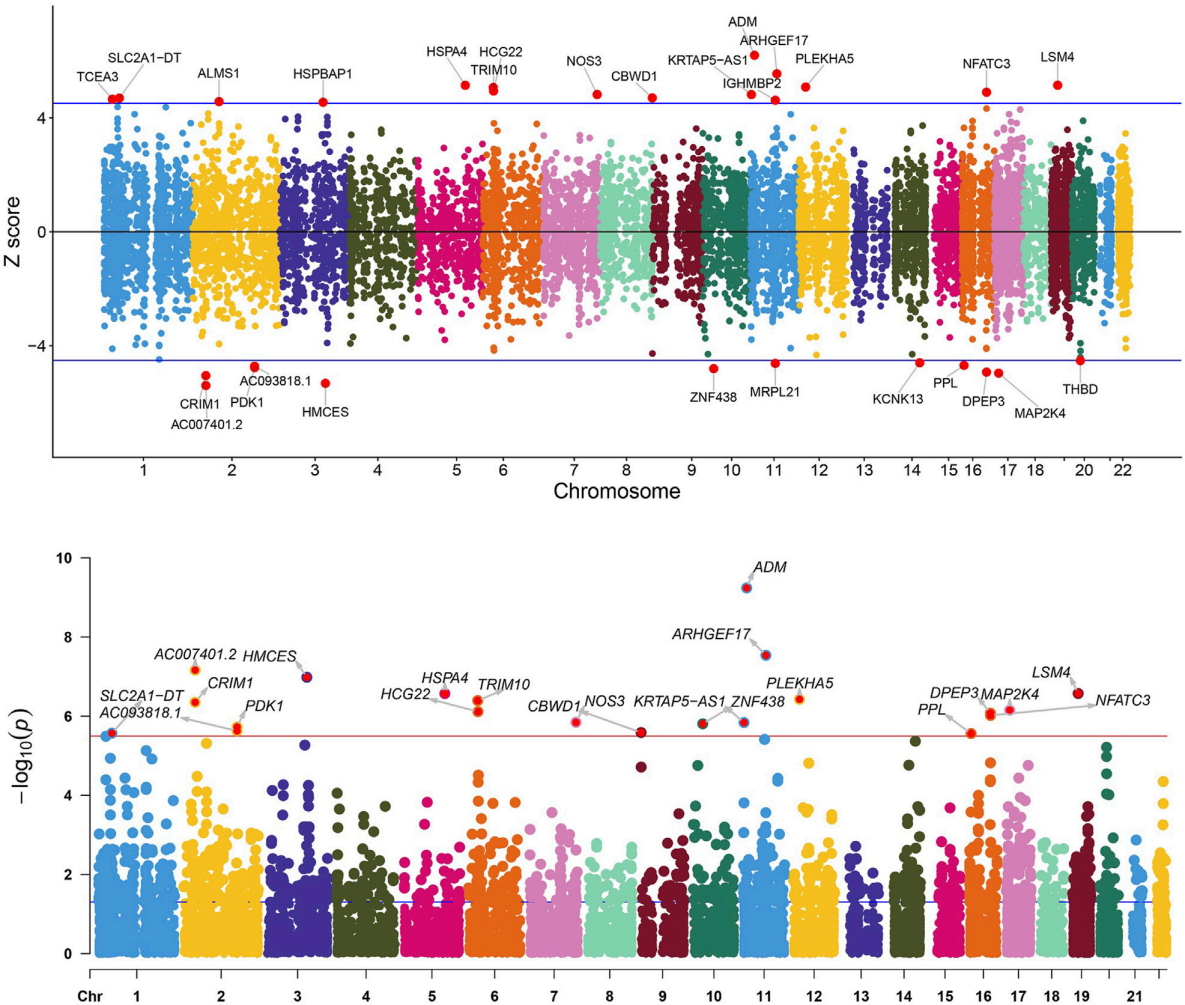
Manhattan plot of the SMR analysis results using QTLs and VV GWAS summary statistics. The red dashed line represents the Bonferroni-corrected significance threshold (Sig_P_Thresh). Z-score filtration was applied, with the blue dashed line indicating the Z score significance level, where Sig_Z_Thresh was calculated as qnorm [1—(0.05/Sig_P_Thresh)].

In the initial phase of our analysis, we conducted SMR analysis using the eQTLGen dataset to identify genes significantly associated with VV features. To establish the significance threshold for the *p* values, we applied the Bonferroni method to establish the threshold for the *p* values. Specifically, for the eQTLGen dataset, we obtained a wealth of information, resulting in 15,633 candidate genes. To maintain a robust level of significance, we set the significance threshold at (0.05/15,633 = 3.198362e-06). In the SMR analysis, we identified 21 genes (*SLC2A1-AS1, CRIM1, PDK1, AC007401.2, AC093818.1, HMCES, HSPA4, TRIM10, HCG22, NOS3, CBWD1, ZNF438, ARHGEF17, ADM, KRTAP5-AS1, PLEKHA5, NFATC3, PPL, DPEP3, MAP2K4,* and *LSM4*) that met the significance threshold of *p* < 3.198362 E-06. To enhance interpretability, Manhattan plots were generated for a clear visualization of the SMR results (Table 1; Figure 2).

**TABLE 1:** SMR/HEIDI results of the GWAS data on VV, blood eQTL data, and colocalization results between GWAS and the blood eQTL data for genes passing the SMR Test.

Ch	Gene	Gene probe	SMR and HEIDI tests					COLOC tests		
			Top SNP	P_GWAS	P_SMR	B_SMR	P_HEIDI	GWAS SNP	PP.H4	N_SNP
		Probe position	SNP position	P_eQTL		SE_SMR	N_HEIDI	SNP position	PP.H3	
1	SLC2A1-AS1	ENSG00000227533	rs3768037	2.38E-06	2.69E-06	0.052	8.64E-01	rs57247989	**0.808**	1,970
		43436874	43412662	0.00E+00		0.011	20	43414370	0.136	
2	CRIM1	ENSG00000150938	rs10172196	1.40E-07	4.45E-07	−0.298	2.70E-01	rs11894187	**0.725**	1,674
		36680673	36780549	7.19E-71		0.059	20	36781708	0.275	
	PDK1	ENSG00000152256	rs2701268	1.41E-06	1.87E-06	−0.188	1.37E-01	rs115108917	1.277E-04	2,365
		173454962	173351199	2.65E-213		0.039	20	173221757	9.999E-01	
	AC007401.2	ENSG00000217075	rs10194100	1.05E-08	6.85E-08	−0.141	3.92E-01	rs11894187	**0.969**	940
		36769179	36783127	9.29E-59		0.026	20	36781708	0.031	
	AC093818.1	ENSG00000225205	rs2701268	1.41E-06	2.35E-06	−0.187	6.45E-01	rs72888132	0.000	2,226
		173383446	173351199	6.65E-117		0.040	20	173226674	1.000	
3	HMCES	ENSG00000183624	rs72981128	3.58E-08	1.04E-07	−0.318	5.59E-02	rs2713575	0.000	2,384
		129011350	128994942	2.72E-92		0.060	20	128294355	1.000	
5	HSPA4	ENSG00000170606	rs72801474	2.00E-07	2.69E-07	0.198	3.17E-01	NA	0.000	2052
		132414897	132444128	1.38E-271		0.038	20	NA	1.000	
6	TRIM10	ENSG00000204613	rs144447022	4.81E-08	4.04E-07	0.375	1.96E-01	rs2524005	**0.971**	558
		30124216	29244219	2.56E-42		0.074	20	29899677	0.029	
	HCG22	ENSG00000228789	rs1265054	4.18E-07	7.69E-07	0.099	7.76E-02	rs1811197	0.090	1,660
		31024447	31079643	7.92E-118		0.020	20	31327660	0.910	
7	NOS3	ENSG00000164867	rs1800781	9.46E-07	1.43E-06	0.200	3.11E-01	rs1800781	**0.997**	1,143
		150699879	150692444	6.09E-155		0.041	20	150692444	0.002	
9	CBWD1	ENSG00000172785	rs3008111	2.10E-06	2.59E-06	0.074	2.20E-01	rs4740661	**0.807**	1,489
		155010	186620	9.74E-272		0.016	20	227621	0.191	
10	ZNF438	ENSG00000183621	rs2994647	7.53E-07	1.56E-06	−0.282	7.64E-02	rs2994647	**0.998**	2,239
		31227214	31275977	5.98E-90		0.059	20	31275977	0.001	
11	ARHGEF17	ENSG00000110237	rs111318327	2.44E-08	2.89E-08	0.104	7.61E-02	rs77981946	0.021	2,148
		73049735	73009929	0.00E+00		0.019	20	72999787	0.827	
	ADM	ENSG00000148926	rs7107290	3.83E-10	5.75E-10	0.169	1.50E-01	rs7122026	0.682	3,118
		10327585	10294772	0.00E+00		0.027	20	10255108	0.318	
	KRTAP5-AS1	ENSG00000233930	rs1809668	1.21E-06	1.45E-06	0.109	9.41E-02	rs7933575	**0.924**	1,934
		1606498	1619907	0.00E+00		0.023	20	93709444	0.075	
12	PLEKHA5	ENSG00000052126	rs1514831	2.79E-07	3.74E-07	0.173	6.70E-02	rs4763522	**0.907**	3,145
		19405991	19303772	3.80E-265		0.034	20	19324343	0.093	
16	NFATC3	ENSG00000072736	rs7204192	5.40E-07	9.58E-07	0.237	8.00E-02	NA	**0.742**	2,490
		68190908	68039309	1.41E-120		0.048	20	NA	0.257	
	PPL	ENSG00000118898	rs12446456	2.04E-06	2.75E-06	−0.185	1.50E-01	rs841217	0.638	1,592
		4971625	4922201	1.06E-190		0.040	20	4746392	0.360	
	DPEP3	ENSG00000141096	rs112310696	6.81E-07	8.38E-07	−0.140	4.21E-01	NA	**0.812**	2,265
		68012149	68038592	0.00E+00		0.028	20	NA	0.187	
17	MAP2K4	ENSG00000065559	rs12451415	1.87E-08	7.03E-07	−0.388	4.18E-01	rs1468501	0.159	624
		11985644	12006780	6.14E-26		0.078	20	11921944	0.841	
19	LSM4	ENSG00000130520	rs73925443	1.75E-07	2.67E-07	0.182	2.08E-01	rs74929147	**0.996**	1,026
		18425562	18429163	2.07E-194		0.035	20	18413061	0.004	

Probe and SNP positions are indicated in GRCh37. Bold numbers denote large PP.H4 (>0.70). Ch represents chromosome; P_GWAS is the *p*-value of the top SNP from the GWAS data; P_eQTL is the *p*-value of the top SNP from the eQTL data; P_SMR is the *p*-value for the SMR test; B_SMR is the effect size from the SMR test; SE_SMR is the standard error of B_SMR; P_HEIDI is the *p*-value for the HEIDI test; N_HEIDL is the number of SNPs used in the HEIDI test; GWAS SNP is the lead variant with the smallest *p*-value from the GWAS data in the region analyzed by the colocalization test (±1 Mb from the GWAS SNP position); N_SNP is the number of SNPs used in the colocalization test.

Following the identification of these significant genes, we employed the HEIDI heterogeneity test (p_HEIDI >0.05) to filter out genes lacking horizontal pleiotropy. In summary, through the integrated analysis of GWAS and blood eQTL data, we identified a total of 21 genes significantly associated with VV features in blood.

**FIGURE 3:**
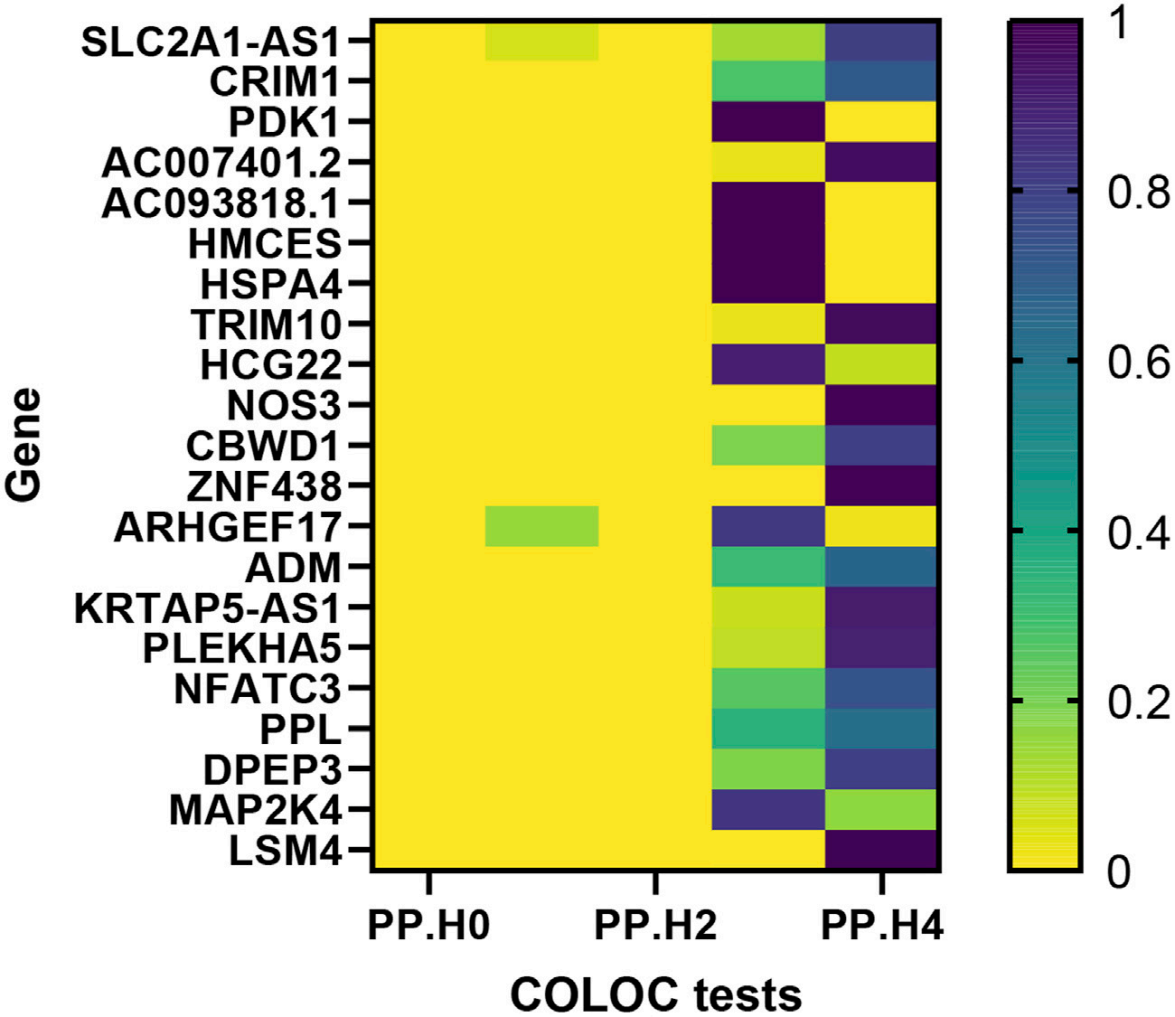
Heat map of the colocalisation analysis results using QTLs and VV GWAS summary statistics. The x-axis represents five hypotheses of colocalization, while the y-axis indicates the genes involved in the analysis. Each colored block in the heat map represents the probability associated with a specific colocalization hypothesis.

**FIGURE 4:**
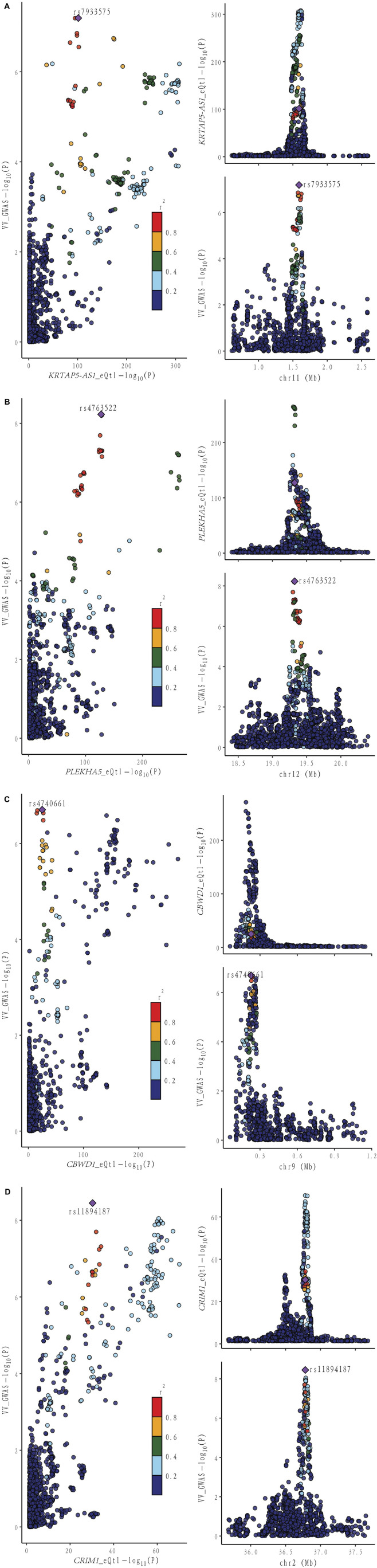
Locus comparison plot illustrating the colocalization analysis results for the single nucleotide variants associated with gene expression in both blood and VV data. Each dot on the plot represents a SNP, and its color corresponds to the LD value (r2) with the GWAS lead variant, depicted as a purple diamond. In the right panel, the genomic positions (in megabases, GRCh37) on the chromosome are presented on the x-axis. The -log10 *p* values for SNPs from the VV GWAS (top) and the eQTL study for the genes (bottom) are displayed on the y-axis. In the left panel, a comparison of *p* values is shown for both the VV GWAS and the gene expression eQTL study. Different genes are displayed separately: **(A)** KRTAP-AS1. **(B)** PLEKHA5. **(C)** CBWD1. **(D)** CRIM1.

Moving forward, we conducted colocalization analysis to integrate the GWAS and blood eQTL data for the genes that successfully passed the SMR test. This analysis aimed to assess whether these genes were colocalized with the trait of interest, VV. The results from the colocalization test provided robust evidence supporting colocalization between the trait and all 12 genes (*KRTAP5-AS1*, *SLC2A1-DT*, *PLEKHA5*, *NOS3*, *LSM4*, *DPEP3*, *CRIM1*, *ZNG1A*, *ZNF438*, *NFATC3*, *TRIM10*, and *AC007401.2*) that met the criteria of both the SMR and HEIDI tests. Therefore, we consider these genes as high-priority candidates for subsequent functional studies (Figures 3, 4).

### 3.2 MR analysis validates potential gene causal relationships

#### 3.2.1 Discovery cohort

Utilizing cis-eQTL data provided by the eQTLGen Consortium and by employing SMR and colocalization analyses, we identified 12 potential genes that could influence VV. Subsequently, a two-sample MR analysis was conducted on the European summary statistics involving patients with VV. In the discovery cohort, which comprised 17,027 patients and 190,028 controls from the FinnGen cohort, the inverse variance weighted (IVW) MR analysis amalgamated the effect estimates from each genetic instrument. The genetically predicted expression of nine genes was found to be associated with VV risk, after multiple testing adjustments (FDR correction). To ensure the robustness of our findings, we performed tests for horizontal pleiotropy, which indicated no evidence of horizontal pleiotropy in the dataset.

In our study, we performed a two-sample MR analysis using the 12 genes. The results are presented in Table 2. In the discovery cohort, significant associations were observed for nine genes (*KRTAP5-AS1, SLC2A1-DT, NOS3, PLEKHA5, LSM4, DPEP3, CRIM1, AC007401.2,* and *ZNG1A*). Heterogeneity tests and horizontal pleiotropy tests were conducted. The heterogeneity test revealed heterogeneity for *PLEKHA5* and *CBWD1*. Consequently, we applied the random effects inverse variance weighted method to address this heterogeneity in the subsequent analysis. The results of the horizontal pleiotropy analysis indicated the absence of horizontal pleiotropy. These additional details provide a nuanced understanding of the robustness and validity of our findings in the context of VV genetics.

**TABLE 2:** MR results for genes expression significantly associated with VV after FDR correction.

Gene	FINNGEN cohort						UK Biobank cohort					
	method	SNPs	OR (95% CI)	IVW P_FDR	MR-Egger intercept	Egger intercept *p*-value	method	SNPs	OR (95% CI)	IVW P_FDR	MR-Egger intercept	Egger intercept *p*-value
KRTAP5-AS1	IVW	36	1.08 (1.05 1.11)	1.42E-10	0.002	0.76	IVW	31	1.002 (1.001 1.003)	6.59E-07	2.10E-05	0.92
PLEKHA5	IVW*	21	1.13 (1.06 1.20)	6.90E-05	0.007	0.33	IVW	12	1.003 (1.001 1.004)	1.13E-02	1.09E-04	0.57
CBWD1	IVW*	16	1.05 (1.01 1.11)	1.42E-02	0.013	0.42	IVW	12	1.001 (1.000 1.002)	1.48E-02	−5.31E-05	0.84
CRIM1	IVW	9	0.87 (0.81 0.95)	3.67E-03	−0.006	0.58	IVW	9	0.997 (0.990 1.000)	4.06E-02	−2.79E-04	0.43

IVW*: Inverse variance weighted (multiplicative random effects).

#### 3.2.2 Replication cohort

In our endeavor to validate the genetic associations identified in the discovery phase, we conducted a rigorous replication analysis using data from the UK Biobank cohort, comprising 10,044 cases and 452,966 controls. This replication effort aimed to reproduce the effect estimates of the top nine genes identified in the discovery stage, providing crucial insights into the robustness and generalizability of our findings.

We observed significant associations with VV for *KRTAP5-AS1* [Odds ratio (OR) = 1.08, 95% CI: 1.05–1.11, *p* = 1.42e-10], *PLEKHA5* (OR = 1.13, 95% CI: 1.06–1.20, *p* = 6.90e-5), *CBWD1* (OR = 1.05, 95% CI: 1.01–1.11, *p =* 1.42e-2), and *CRIM1* (OR = 0.87, 95% CI: 0.81–0.95, *p =* 3.67e-3). Notably, these findings underscore the robustness and consistency of the genetic associations across different cohorts, reinforcing the potential relevance of these genes to VV.

Of particular significance, our replication analysis demonstrated successful replication beyond the FDR method for four genes (*KRTAP5-AS1, PLEKHA5, CBWD1,* and *CRIM1*), exhibiting 100% consistency in the direction of the effect. This remarkable consistency further validates the genetic associations and supports the reliability of our findings. Importantly, horizontal pleiotropy tests indicated no evidence suggesting the presence of this confounding factor in the data, further bolstering the robustness and validity of our replicated genetic associations with VV.

### 3.3 Validation

#### 3.3.1 TWAS analysis validates transcriptome-level causal relationships

In our pursuit of strengthening the causal inference and gaining deeper insights into the genetic associations with VV, we conducted a TWAS analysis on the four genes identified in the previous analyses. This comprehensive approach aimed to elucidate the transcriptional level associations of these genes with VV and to bolster the evidence supporting their potential causal role. Additionally, we performed colocalization analysis to further validate the potential causal relationship between these genes and VV.

The results revealed significant associations at the transcriptional level for the four genes with VV, with all colocalization probabilities (PP.H4) exceeding 0.7. Specifically, elevated transcription levels of *KRTAP5-AS1* (Z score = 4.896, *p =* 9.76e-7), *PLEKHA5* (Z score = 5.205, *p =* 1.94e-7), and *CBWD1* (Z score = 4.839, *p =* 1.30e-6) were significantly correlated with an increased risk of VV, whereas increased transcription level of *CRIM1* (Z score = −3.629, *p =* 2.85e-4) was significantly associated with a decreased risk of VV. Notably, these TWAS findings were consistent with those from the SMR analysis, providing robust support for the transcriptional associations of these genes with VV.

We also conducted a TWAS colocalization analysis and found that the probability of these four genes sharing the same causal variant (PP.H4: *KRTAP5-AS1* = 0 .943, *PLEKHA5* = 0.993, *CBWD1* = 0.802, *CRIM1* = 0.932) exceeded the predefined threshold (*p* > 0.7).This robust result unequivocally strengthens the indication of the causal relationship between these four genes and VV, further solidifying the genetic basis of VV and providing crucial insights into the potential mechanisms underlying this condition (Table 3).

**TABLE 3:** TWAS results between GWAS and blood eQTL data for genes through the TWAS/Colocalization Test.

GENE	HSQ	GWAS.ID	GWAS.Z	EQTL.ID	EQTL.R2	EQTL.Z	EQTL.GWAS.Z	NSNP	NWGT	MODEL	MODELCV.R2	MODELCV.PV	TWAS.Z	TWAS.P	COLOC.PP4
KRTAP5-AS1	0.1237	rs7933575	5.41	rs10768561	0.095857	8.03	4.68303	358	6	lasso	0.11	2.3E-16	4.89637	9.76E-07	0.943
PLEKHA5	0.081	rs969235	−5.41	rs1514831	0.0562	−6.14	−5.13701	442	442	susie	0.057	6.3E-09	5.20522	1.94E-07	0.993
CBWD1	0.2218	rs526509	5.15	rs478882	0.151	9.3	4.360937	452	26	enet	0.2	1.2E-28	4.839414	1.30E-06	0.802
CRIM1	0.0805	rs13406184	5.72	rs10197570	0.0239	−5.02	4.66795	672	672	susie	0.037	0.0000026	−3.62859	2.85E-04	0.932

HSQ stands for gene heritability. GWAS.ID and GWAS.Z are the top GWAS SNPs rsIDs and Z-scores. EQTL.ID and EQTL.R2 are the best eQTLs rsIDs and cross-validated *r* ^2^. NSNP is the locus SNP count. MODEL is the best-performing model. TWAS.Z and TWAS.P are key TWAS statistics: Z score and *p*-value. COLOC.PP4 represents the colocalization analysis results.

#### 3.3.2 GWAS analysis validates genome-level causal relationships

We also conducted a GWAS analysis using MAGMA software on the quartet of genes highlighted in preceding studies. This approach aimed to illuminate the associations of these genes with VV in genome-level, thereby reinforcing the evidence underpinning their potential causal involvement.

The outcomes unveiled noteworthy genome-level associations for the aforementioned genes with VV. Specifically, heightened transcription levels of *KRTAP5-AS1* (*p* = 0.022), *PLEKHA5* (*p* = 3.34E-06), and *CBWD1* (*p* = 0.001) exhibited significant correlations with an elevated VV risk, whereas increased transcription levels of *CRIM1* (*p* = 0.008) were significantly linked with a reduced VV risk (Table 4). Importantly, these GWAS findings corroborated those from the SMR analysis, thereby fortifying the robustness of the transcriptional associations of these genes with VV.

**TABLE 4:** GWAS result based on MAGMA.

Gene	Ch	Position	NSNPS	NPARAM	N	ZSTAT	P
KRTAP5-AS1	11	1606498	32	10	207055	2.007	**0.022**
PLEKHA5	12	19405991	1,144	90	207055	4.504	**3.34E-06**
CBWD1	9	155010	171	28	207055	3.105	**0.001**
CRIM1	2	36680673	994	94	207055	2.423	**0.008**

Gene: Gene ID from GWAS. Ch: Chromosome of the gene. Position: Annotation boundaries on chromosome. NSNPS: Number of SNPs annotated to the gene after QC. NPARAM: Relevant parameters in the model (approximate for SNP-wise models, based on retained principal components for PCA, mean NPARAM for multimodels). N: Sample size for gene analysis (may vary for allosomal chromosomes or due to variable SNP sample size). ZSTAT: Z-value for gene association (based on permutation *p*-value). P: Gene *p*-value (asymptotic sampling distribution).

### 3.4 DGIdb screening for pharmacologically relevant genes

Incorporating the comprehensive resources provided by the DGIdb database, our analysis identified four pharmacologically relevant genes (*KRTAP5-AS1, PLEKHA5, CBWD1,* and *CRIM1*). This crucial step has unveiled potential drug targets with direct relevance to the treatment of VV, shedding light on promising avenues for targeted therapeutic interventions. By leveraging this pharmacogenomic information, we have advanced our understanding of the molecular underpinnings of VV and have laid the groundwork for the exploration of novel therapeutic strategies tailored to the genetic landscape of this condition.

## 4 Discussion

To study the potential pathogenic genes of VV and to search for new drug targets, we used an integrated analysis method combining SMR analysis and colocalization analysis. We used an eQTL meta-analysis dataset based on peripheral blood samples as the exposure data with a relatively large sample size for the outcome data from two large databases, FinnGen and UK Biobank. After the analysis of the preliminary results, we screened out some candidate genes and identified them using the DGIdb database. Finally, four VV-related genes were identified: *KRTAP5-AS1*, *PLEKHA5, CBWD1*, and *CRIM1*. The results suggested that there was a potential causal relationship between VV and these four genes. The increased expression of *KRTAP5-AS1, PLEKHA5,* and *CBWD1* was associated with increased risk of VV, and the increased expression of *CRIM1* was associated with a significantly decreased risk of VV. The results of this study may provide important evidence for drug development and individualized treatment of VV and contribute to improving the quality of life of VV patients.

There are few studies on *KRTAP5-AS1* and *CBWD1*. The research on *KRTAP5-AS1* is mainly focused on its long-chain non-coding RNA (lncRNA), which is a kind of conserved RNA with a length of more than 200 nucleotides and no significant protein-coding ability. There is evidence that it can be used as a new biomarker to predict the prognosis of various cancers (You et al., 2018). For example, Song et al. found that *KRTAP5-AS1* can exert the role of a cRNA in regulating claudin-4, and that *KRTAP5-AS1* can regulate *CLDN4* expression as a competitive endogenous RNA of miR-596 and miR-3620-3p. *CLDN4* has been shown to alter expression patterns in various types of cancer, including gastric, pancreatic, and ovarian cancer (Song et al., 2017). In addition, studies have used *KRTAP5-AS1* as a lncRNA signal to predict the prognosis of papillary thyroid cancer (You et al., 2018). Other studies have found the potential of eight lncRNA markers, including *KRTAP5-AS1*, as independent prognostic biomarkers in HBV-positive hepatocellular carcinoma patients (Zhao et al., 2020). Based on the above evidence, we hypothesized that *KRTAP5-AS1* could play a regulatory role as ceRNA, affecting the expression of its neighboring genes or negatively regulating the expression of miRNA target genes as miRNA to improve the risk of varicose veins.

*CBWD1* is located at 9p24.3:121038-179075 (GRCH37/HG19) and located near the 9p telomere. The absence of 9p syndrome can lead to facial deformity, hypotonia, and intellectual disability. Researchers have found that *CBWD1* is associated with some cases of congenital kidney and urinary tract abnormalities. The results have suggested that *CBWD1* is associated with the development of ureteric buds into the urinary tract, and homozygous deletion involving *CBWD1* can manifest as renal hypoplasia (Kanda et al., 2020). Similar to *KRTAP5-AS1*, there is an association between *CBWD1* and cancer, as reported by Wang et al. (2022), who constructed a brisk tmmodel of *CBWD1* as a pivotal gene to predict survival in patients with ovarian cancer. There is evidence that variants on the *CBWD1*/*DOCK8* locus (9P22.3) have enhancer histone markers of *CBWD1*, promoter histone markers, TF-binding motifs, and cis-EQTL in lung, pancreas, liver, fat, musculoskeletal, artery, heart, fibroblasts, blood, and other tissues (Feitosa et al., 2022). This suggests that variations in *CBWD1* may affect the normal function of musculoskeletal, fibroblasts, heart, and blood, thereby causing abnormalities at the venous end and increasing the risk of varicose veins. However, as no such reported cases have been found, further experiments are needed to confirm this conjecture.

*PLEKHA5* and *CRIM1* are two relatively popular genes that have been studied extensively. *PLEKHA5* belongs to the *PLEKHA* family and contains the pleckstring homology domain. The domain is thought to mediate phosphatidylinositol binding properties and is associated with multiple intracellular functions, such as signaling, cytoskeleton rearrangement, membrane protein targeting, and vesicle trafficking (Dowler et al., 2000). *PLEKHA5* has been shown to be expressed on the plasma membrane and associated with microtubules, and it has also been demonstrated to play an important role in cell migration by wound healing assay experiments (Zou and Zhong, 2012). To date, the most thoroughly studied aspect of *PLEKHA5* has been its potential regulatory role in melanoma brain metastasis. *PLEKHA5* is associated with brain cell activity, and decreased expression of *PLEKHA5* inhibited the proliferation of brain nutrient cells as well as the migration of pro-brain cells in an *in vitro* blood-brain barrier model. *PLEKHA5* expression in melanoma is associated with the early development of brain metastases, and up to 75% of patients with stage IV melanoma develop central nervous system metastases during the course of the disease, according to a study by Jilaveanu et al. (2015). *PLEKHA5* has also been reported to be involved in the central nervous system homing mechanism in metastatic disease, and inhibition of *PLEKHA5* may reduce the passage across the blood-brain barrier and reduce the proliferation and survival of melanoma cells in the brain and extrabrain regions (Jilaveanu et al., 2015). Since there is no experimental evidence to support this, we can only speculate that *PLEKHA5*’s positive causal relationship to VV may also be mediated by brain metastasis. In addition to melanoma, *PLEKHA5* has also been associated with gastric cancer, with studies finding that tyrosine phosphorylation of *PLEKHA5* is *MET*-dependent and associated with *MET* expression and phosphorylation. *PLEKHA5* is able to regulate the survival and peritoneal dissemination of diffuse gastric cancer cells through *MET* gene amplification (Nagamura et al., 2021). In summary, *PLEKHA5* is closely related to multiple internal functions of cells and plays an important role in cell migration, so it may cause varicose veins by promoting the migration of vascular smooth muscle cells.

*CRIM1* is a gene encoding a cysteine-rich repeat protein that is developmentally regulated and involved in vertebrate central nervous system development and organogenesis (Kolle et al., 2000). Evidence that *CRIM1* haploinsufficiency leads to defects in eye development in humans and mice suggests *CRIM1* as a potential causative gene of Macrophthalmia, colobomatous, with microcornea (MACOM) and underscores the importance of *CRIM1* in eye development (Beleggia et al., 2015). In addition, *CRIM1* is also associated with the formation and maintenance of blood vessels *in vivo*. During angiogenesis, *CRIM1* is strongly upregulated in endothelial cells and expressed by a variety of cell lines with adhesion growth, and the formation of capillary structures is impaired in endothelial cells that are transfected *in vitro* (Glienke et al., 2002). We hypothesize that the increased expression of *CRIM1* might reduce the risk of VV because the endothelial structure of blood vessels is not susceptible to damage. We hypothesized that upregulated expression of *CRIM1* reduces the risk of varicose veins because VV is associated with disruption of normal angiogenesis (Chang et al., 2009), however, the endothelial cell structure of blood vessels was less damaged after the gene expression was upregulated, thus reducing the risk of varicose veins.

In the existing research on *PLEKHA5* and *CRIM1*, we did not find any studies linking *PLEKHA5* and *CRIM1* with VV. Our experimental results demonstrate the existence of a potential causal relationship between *PLEKHA5* and *CRIM1* and VV, but the possible pathways or mechanisms are unknown. In the past, dissection of the great saphenous vein and the small saphenous vein, which often occur in VV, were the main treatment. However, this method was risky, and the recurrence rate was relatively high, and up to 24% of patients required additional treatment. As a result, minimally invasive procedures such as radiofrequency ablation, intravenous laser therapy, ultrasound-guided foam sclerosis, and others started to be favored (Nijsten et al., 2009). Minimally invasive surgery has also been shown to be relatively safe with few serious complications (Al Samaraee, McCallum, and Mudawi, 2009). In recent years, many articles have discussed and compared these minimally invasive procedures, for example, researchers have found that in many studies, EVLT appears to be more effective than RFA (Ahadiat et al., 2018). In addition, studies have proposed an alternative method of varicose vein implantation, the reduced sine microchannel (DSMC), which can maintain good blood flow in the human leg and avoid tissue damage and other problems (Afzal et al., 2018).

At present, most studies on VV are investigation, descriptive clinical studies and randomized clinical trials, and few studies analyze the pathogenesis and treatment of VV at the genetic level. However, we used the latest GWAS data, combined with the information of eQTL database and DGIdb database. The original data were screened and verified strictly to ensure the accuracy and robustness of the results. Our study also used a comprehensive analysis method including MR Analysis to avoid various biases that may exist in observational epidemiological studies. In addition, different from previous studies that used proteins as exposure factors, we use genes with stable genetic characteristics that are not affected by environmental factors as exposure factors, which can more intuitively and accurately reflect the causal relationship between genes and VV. The four genes we identified have the potential to be targeted pharmacologically and could serve as therapeutic targets in future VV therapies. The current VV treatment is mainly surgical. Although the safety of minimally invasive surgery has been improved, the existence of surgical complications still cannot be completely avoided. Our research findings have contributed to the pharmacological treatment of VV. At the same time, our results provide a new idea and direction for the study of the pathogenesis of VV. In the future, we will continue to conduct more in-depth research and experiments on these four genes, verify and analyze the above conjecture, and strive to give a reasonable explanation of the pathogenesis of varicose veins at the gene level.

There are some limitations in this study. First, our study was only related to theoretical drug target discovery. Second, the two-sample MR analysis used in the study was based on publicly available and free GWAS aggregates with limitations in updating, selection, and screening of the data. Due to the limitations of sample type and public databases, we could not further conduct subgroup analysis on sex differences in varicose veins. The samples included in this study were from a European population. MR can effectively reduce the statistical bias caused by population differences, but the results still may not be generalizable to non-European populations. In addition, although we used a variety of methods to validate the results of our experiments, we cannot rule out the possibility that other confounding factors that are not considered may have influenced the effects of the genes on the disease. Further exploration and validation are required.

In conclusion, this study identified the effects of *KRTAP5-AS1, PLEKHA5, CBWD1,* and *CRIM1* genes on VV. No previous study has found a potential causal relationship between these four genes and VV. Our results provide new causal evidence for VV-related genes as well as potential drug targets and therapeutic ideas for VV therapy.

## Data Availability

The original contributions presented in the study are included in the article/Supplementary material, further inquiries can be directed to the corresponding authors.
